# Coconut oil: an overview of cardiometabolic effects and the public health burden of misinformation

**DOI:** 10.20945/2359-3997000000641

**Published:** 2023-06-19

**Authors:** Bernardo Frison Spiazzi, Ana Cláudia Duarte, Carolina Pires Zingano, Paula Portal Teixeira, Carmen Raya Amazarray, Eduarda Nunes Merello, Laura Fink Wayerbacher, Laura Penso Farenzena, Poliana Espíndola Correia, Marcello Casaccia Bertoluci, Fernando Gerchman, Verônica Colpani

**Affiliations:** 1 Universidade Federal do Rio Grande do Sul Programa de Pós-graduação em Ciências Médicas: Endocrinologia Porto Alegre RS Brasil Programa de Pós-graduação em Ciências Médicas: Endocrinologia, Universidade Federal do Rio Grande do Sul, Porto Alegre, RS, Brasil; 2 Universidade Federal do Rio Grande do Sul Faculdade de Medicina Departamento de Medicina Interna Porto Alegre RS Brasil Departamento de Medicina Interna, Faculdade de Medicina, Universidade Federal do Rio Grande do Sul, Porto Alegre, RS, Brasil; 3 Hospital de Clínicas de Porto Alegre Divisão de Endocrinologia e Metabologia Porto Alegre RS Brasil Divisão de Endocrinologia e Metabologia, Hospital de Clínicas de Porto Alegre, Porto Alegre, RS, Brasil

**Keywords:** Coconut oil, social media, misinformation, internet, saturated fatty acids

## Abstract

Recent data from meta-analyses of randomized clinical trials (RCTs) suggest that dietary intake of coconut oil, rich in saturated fatty acids, does not result in cardiometabolic benefits, nor in improvements in anthropometric, lipid, glycemic, and subclinical inflammation parameters. Nevertheless, its consumption has surged in recent years all over the world, a phenomenon which can possibly be explained by an increasing belief among health professionals that this oil is as healthy as, or perhaps even healthier than, other oils, in addition to social network misinformation spread. The objective of this review is to present nutritional and epidemiological aspects related to coconut oil, its relationship with metabolic and cardiovascular health, as well as possible hypotheses to explain its high rate of consumption, in spite of the most recent data regarding its actual effects.

## INTRODUCTION

Coconut oil was an unusual component of westernized dietary patterns until the mid-2010s. In spite of being rich in saturated fatty acids, there has been a surge in beliefs that coconut oil is healthy and possibly superior to other oils in improving cardiometabolic outcomes (1), without support from strong scientific evidence (2-5). This phenomenon has been accompanied by a rise in consumption rates in the last decade (6) and might be explained by the spread of misinformation in internet-based and traditional media (7,8). This review aims to summarize the current knowledge on coconut oil's general characteristics, its effects on cardiometabolic and anthropometric markers, and the possible role of misinformation spread as the catalyst for the observed change in dietary patterns in the West.

## COCONUT OIL: CHARACTERISTICS AND METABOLISM

**Figure 1:**
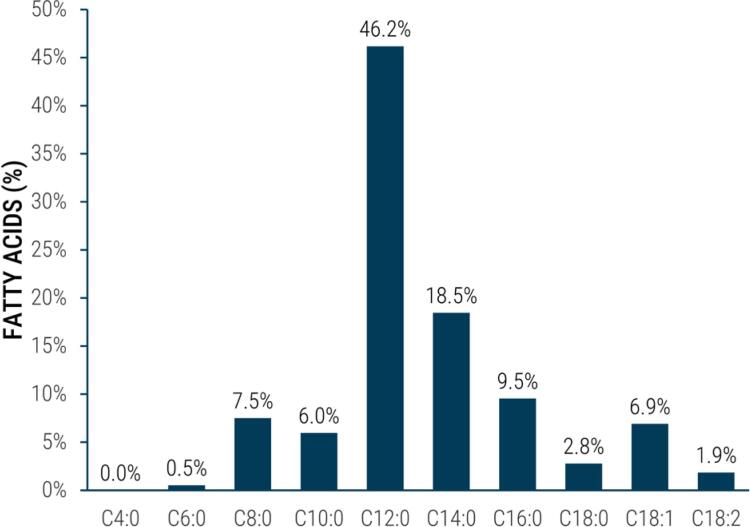
Coconut oil fatty acid composition. C4:0 = butyric; C6:0 = caproic acid; C8:0 = caprylic acid; C10:0 = capric acid; C12:0 = lauric acid; C14:0 = myristic acid; C16:0 = palmitic acid; C18:0 = stearic acid; C18:1: = oleic acid; C18:2 = linoleic acid.

Approximately 91% of coconut oil fatty acids (FAs) are saturated (SFAs), of which 46.2% are lauric acid (C12:0), 18.5% myristic acid (C14:0), 9.5% palmitic acid (C16:0), 7.5% caprylic acid (C8:0), and 6% capric acid (C10:0). The remaining FAs are monounsaturated (MUFAs) (7%) and polyunsaturated (PUFAs) (2%). Coconut oil is low in linoleic acid (18:2) and contains no linolenic acid (18:3) [both essential fatty acids] (Figure 1) (9).

Dietary fats are composed of a mixture of fatty acids (SFAs, MUFAs, and PUFAs), and their composition varies according to their source: animal or vegetable. Fats with a higher proportion of MUFAs and PUFAs are generally liquid at room temperature and are referred to as “oils,” whereas fats with a higher proportion of SFAs, especially long-chain FAs, are usually solid at room temperature and are called “fats” (10,11). Hence, “coconut oil,” as it is popularly known, is not the most correct term, since it is solid at room temperature precisely due to its high amount of SFAs and is, therefore, considered a solid fat for nutritional purposes (11).

Lauric acid, the main FA contained in coconut oil, can be classified as both a medium-chain fatty acid (MCFA) and a long-chain fatty acid (LCFA); thus, it is a FA with intermediate properties (12). MCFAs, mainly composed of caprylic (C8:0) and capric (C10:0) acids, are absorbed in the small intestine bound to albumin and reach the liver via the portal system without increasing triglyceridemia (13). Conversely, 70% to 75% of lauric acid is absorbed within chylomicrons (similarly to LCFA absorption) (6), and its presence in chylomicrons is dose-dependent (14). Overall, MCFAs (C8:0 and C10:0) have a low molecular weight (512 on average), unlike LCFAs, which have a higher molecular weight (the molecular weight of coconut oil is 638). FAs with lower molecular weights facilitate pancreatic lipase action, being hydrolyzed more efficiently in the small intestine compared to FAs with longer chains (15).

LCFAs are esterified in enterocytes, where they form triglycerides (TGs) that are transported to the bloodstream via the lymphatic system by chylomicrons. Lipoprotein lipase hydrolyzes chylomicron TGs, releasing FAs to peripheral tissues, which can either be used as an energy source or be re-esterified into new TGs for storage (10). LCFAs require the transporter carnitine palmitoyl transferase in the outer mitochondrial membrane to be internalized into the organelle and oxidized to acetyl-CoA to serve as an energy source (16,17). By contrast, MCFAs are absorbed directly into the bloodstream and are not significantly incorporated in chylomicrons and very-low-density lipoproteins (VLDL). Hence, they are considered a quick source of energy, because, as they pass through the enterocytes, MCFAs reach the portal circulation and are transported to the liver bound to albumin, where they are oxidized. It has been speculated that, due to their rapid metabolism, MCFAs could stimulate thermogenesis, decreasing their deposition in adipose tissue, which would increase satiety without increasing serum total cholesterol (TC) levels (10). However, there are no experimental studies that prove these theories, and even if there were, these findings could not be extrapolated to coconut oil due to its high concentration of lauric acid, which is not absorbed, transported, and metabolized in this manner.

## COCONUT OIL: MARKET AND CONSUMPTION

Coconut products are now part of people's diets worldwide, although only some Asian populations, such as Sri Lankans, Minangkabau, and Filipinos, have coconut as part of their daily diet. As expected, the Asian continent holds the largest area of coconut tree plantations, where coconut palm cultivation is intended for commercial fruit copra (dried coconut meat) exploitation for oil production and dehydrated dried coconut (18). The Philippines, Indonesia, and India are the three most important coconut oil producers worldwide (19).

In the past few years, international coconut oil consumption and production, especially that of virgin unrefined coconut oil, has increased significantly (6). It is estimated that 640,000 tons of coconut oil were consumed in the Philippines, 449,000 tons in India, and 468,000 tons in the United States in 2016 (20). The same is observed in Europe, where the United Kingdom stands out as one of the main importers of coconut oil, having been responsible for around 7% of Sri Lanka's coconut exports in 2015 (21). There are no records regarding Brazilian consumption of coconut oil until 2020, when an online survey of graduate students and laymen in southern Brazil found that 59.1% of the responders consumed coconut oil (22). However, its use for food preparation or as a dietary supplement was uncommon until the mid-2010s. As it is rich in SFA, a type of fat well known as being related to increased plasma levels of low-density lipoprotein (LDL-C) and increased cardiovascular risk, the consumption of coconut oil was not recommended by scientific societies (10).

**Figure 2:**
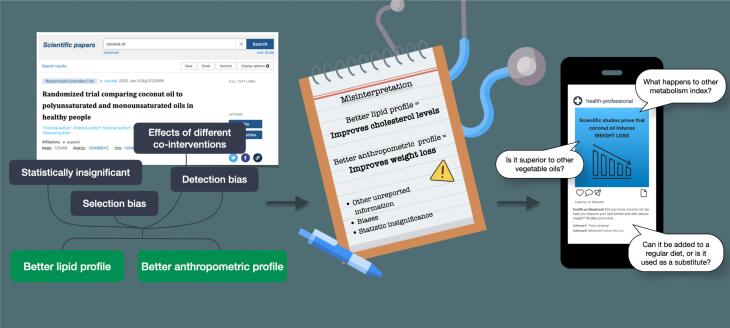
Model of the process that pervades coconut oil misinformation generation originating from inadequate interpretation of scientific evidence. The model demonstrates the steps which were likely taken to result in an increased dietary consumption of coconut oil. These steps occurred after publication of studies that were typically conducted with methodological limitations and biases (and can possibly also be applied to explain the emergence of health misinformation in general). This can result in a skewed interpretation of scientific data, subsequently leading to inappropriate clinical applications and triggering health care-related misinformation spread on social media.

Human studies testing the effects of coconut oil on cardiometabolic health date back to the 1990s (23,24), but it was only after the publication and possibly dissemination of the results of other studies (25,26) at scientific meetings and on social networks that coconut oil sparked people's interest as a new option for consumption and for healthy diet prescriptions. These short-term studies showed a slight improvement in lipid and anthropometric profiles with coconut oil intake in healthy adult populations (25,26). However, their results were based on surrogate markers of cardiometabolic health, and no studies to date have actually evaluated the potential benefit of coconut oil consumption in preventing diabetes and hard cardiovascular outcomes. Furthermore, it is also possible that these studies set off an interest in the food industry to market coconut oil as a healthy food by broadcasting its benefits on websites, blogs, health professionals’ social media profiles, and in clinical practice, spreading the news that coconut oil is healthy to cook with or to add to salads and other meals (Figure 2) (1).

## COCONUT OIL: EFFECTS ON CARDIOMETABOLIC PARAMETERS

Some Asian populations, such as Sri Lankans, Minangkabau, Filipinos, Pukapukan, and Tokelauan, had lower cholesterol levels and lower rates of cardiovascular disease than westernized populations before they were introduced to a more westernized diet (27-30). Among the possible hypotheses proposed to explain this phenomenon, it was suggested that their high rate of consumption of coconut oil might have been responsible for these findings. However, it is worth noting that even though the main source of FAs in these populations’ diet was indeed coconut fat – unlike more westernized populations, which have greater consumption of animal fat – their greatest source of protein was fish, and their major source of carbohydrates was native fruits. Hence, their dietary pattern was healthier overall, richer in high-fiber foods, and lower in ultra-processed foods and sucrose (29).

**Figure 3:**
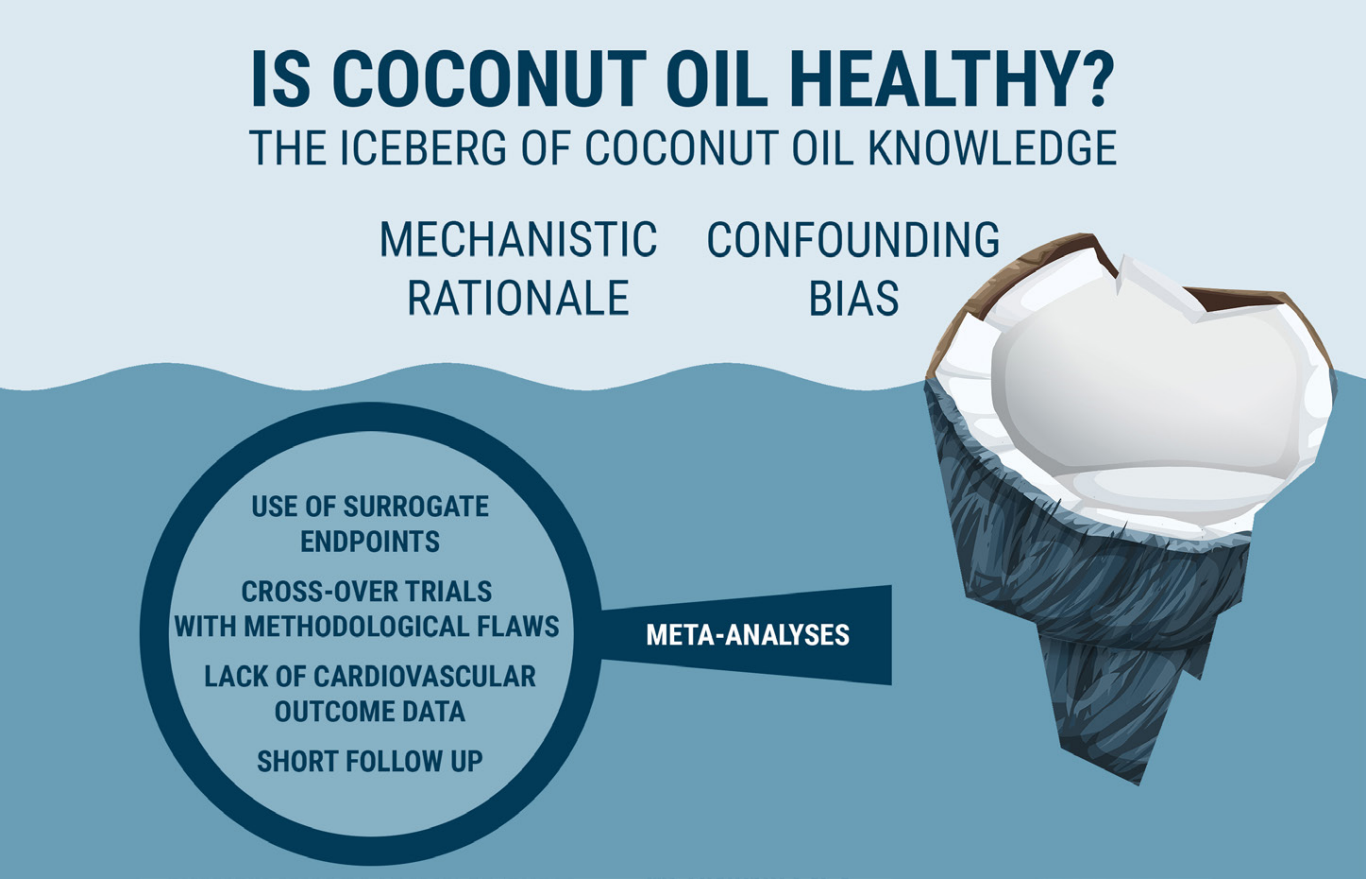
Conceptual framework of coconut oil knowledge distribution. Iceberg model on coconut oil knowledge distribution. Misinformation is generated when looking only at the “surface” concepts, failing to acknowledge the information presented “underwater”.

Coconut oil intake is associated with worsening lipid profiles and increasing concentrations of plasma LDL-C, a well-defined risk factor for cardiovascular disease, while providing no improvement in body weight, glycemic control, and inflammatory parameters compared to other non-tropical oils (2-4). There are several methodological limitations in the intervention studies that evaluated the benefit of coconut oil to metabolic parameters (Figure 3). In three meta-analyses that evaluated the consumption of coconut oil versus other oils in relation to lipid, anthropometric, inflammatory markers, and blood glucose levels, cross-over studies were included (2-4). Among the limitations related to these studies are the lack of a definition regarding the wash-out period and the order of randomization of the intervention groups, in addition to the lack of clarity regarding the existence of clinical, nutritional, and laboratory evaluation parameters before and shortly after all interventions. In a recent updated meta-analysis in which these issues were addressed, the results were overall similar to those of previous meta-analyses; however, the consumption of coconut oil did not change LDL levels compared to other oils (5). Furthermore, the randomized clinical trials (RCTs) included in these meta-analyses (2-5) generally had a short follow-up time (1 to 12 weeks) and multiple control arms consisting of oils with different nutritional properties (e.g., chia, safflower, and soybean oils), which may limit the ability to meta-analyze the data. Studies also had different doses and percentages of energy intake from coconut oil, varying from 2% to 25% of daily calories, which means that, in some studies, the amount of SFAs greatly surpassed the maximum daily recommendations for a healthy diet (11).

On the other hand, an Indian RCT followed up 198 people with cardiovascular disease for 2 years and analyzed their metabolic parameters by comparing groups with 15% of their daily caloric intake from coconut oil versus sunflower oil in food preparation. No differences were found in the levels of TC, LDL-C, high-density lipoprotein (HDL-C), TGs, and VLDL; weight; waist circumference; body fat percentage; and glycated hemoglobin (HbA1c) levels between both groups (31). These data suggest that longer term studies are better suited to demonstrate that no benefit was observed with coconut oil consumption in long-term metabolic parameters.

In addition, underpinning medical conduct based on surrogate outcomes has applicability limitations for decision making, often proving to be an inadequate course of action. For instance, it is worth mentioning the study that evaluated the effectiveness of the cholesteryl ester transfer protein (CETP) inhibitor, a drug that significantly increases HDL-C levels, yet was associated with a greater risk of cardiovascular outcomes in its cardiovascular safety study and was precociously interrupted (32).

Furthermore, an observational study has shown that replacing 5% of energy intake from SFAs with the same intake of PUFAs or MUFAs is associated with a 27% and 13% lower risk of mortality, respectively (33). These data corroborate the recommendations of the current dietary guideline for Americans, which recommends reducing SFAs to less than 10% of daily calories and replacing them with unsaturated fatty acids (UFAs) (11). In addition, recent data from long-term prospective cohorts and meta-analyses have shown that these recommendations are associated with prevention of weight gain, reduced insulin resistance and risk of diabetes (34-37).

Moreover, not only do several scientific entities not recommend the use of coconut oil as a preferred FA for human consumption (11,38,39), but also different specialized societies dedicated to metabolic health have taken a stand against the use of coconut oil, especially for weight loss, as there is no scientific evidence in the literature that it has any mechanism which could indicate a potential for this purpose (40).

Considering the fatty acid composition of coconut oil, would it be possible for this food to have antioxidant properties and for its consumption to improve inflammatory parameters? To date, studies that have evaluated the antioxidant effects of coconut oil are mostly preliminary and experimental, and their data cannot be replicated in humans (38). However, virgin coconut oil, extracted by wet-processing directly from coconut milk in a controlled temperature environment, seems to have better nutritional properties, since it retains a greater number of unsaponifiable components, such as vitamin E and polyphenols, thus having greater antioxidant capacity (36). Nonetheless, coconut oil is usually obtained by a dry process from copra oil: it is extracted from the coconut “meat,” which is grated, ground, and cooked in water to extract the oil, and then exposed to very high temperatures or to light for several days until excess moisture is removed. This exposure to sunlight or high temperatures could inactivate known antioxidant components such as tocopherols, tocotrienols, and polyphenols (41).

It is also worth noting that, among all SFAs, lauric acid has the greatest inflammatory potential (42). Studies show that lauric acid is able to activate inflammatory pathways through toll-like receptor (TLR) 4 activation, which leads to secretion of inflammatory cytokines and T-cell activation (37,41). An in vitro study with macrophages observed that lauric acid induces the activation of nuclear factor kappa B (NF-κB), leading to an increase in the expression of cyclooxygenase-2 (COX2) through activation of TLR 2 and 4 (43). A recent RCT meta-analysis comparing the effects of consuming coconut oil rich in lauric acid with other oils found no difference in plasma concentrations of C-reactive protein, an important marker of subclinical inflammation (2). In another RCT, conducted in normocholesterolemic subjects from Malaysia with a 5 week follow-up period, the effects of coconut oil consumption against palm and olive oil consumption on plasma homocysteine concentrations and inflammatory markers, such as tumor necrosis factor-α, interleukin-1β, interleukin-6, interleukin-8, and interferon-γ, were compared, and no difference was found in these parameters among the three groups (44). These data do not support beneficial claims related to coconut oil on different metabolic parameters associated with the development of obesity, diabetes, and cardiovascular disease.

## HEALTH MISINFORMATION

In the last 10 years, coconut oil consumption has been encouraged by health professionals on broad communication media platforms (blogs, websites, social media, the radio, and television), despite the lack of robust evidence (2-5) and recommendations from scientific societies (11,38-40).

Health misinformation is a worldwide issue, and its prevalence increases with mass media or social network production of health content, ultimately expanding the reach of non-evidence-based recommendations to the broader public (7,8).

Social media are essential for information flow, knowledge buildup, and opinion dissemination. Nonetheless, ever since the internet has become part of our everyday life, people have become used to accessing health information easily on their computers and smartphones, although most information comes from unreliable sources. Studies show that information published on health-related websites may present inaccuracies or may contain statements based on low quality evidence (45-49) inasmuch as anyone can publish online (48). Hence, much information – not necessarily knowledge – is being disseminated (50). In fact, not only with coconut oil, but also with other aspects of our daily food intake, we do not know the epidemiology of misinformation and how this impacts our health.

Although social media plays a fundamental role in propagating scientific discoveries in health by presenting facts and findings in an accessible way, it has also paved the way for circulation of unproven information or fallacious conclusions drawn by distorting scientific publications. In this way, the internet has proven to be a fertile ground for rumors, fake news, and unscientific opinions based on beliefs that almost instantly grow and spread, fabricating unfounded theories and false truths which can negatively impact people's health.

In 2002, Eysenbach introduced the term “infodemiology” as the process that enables the identification of knowledge gaps between the best scientific evidence, as proposed by experts in their field, and what most people do or believe (51). In coconut oil's case, these gaps in knowledge were likely generated by mistranslating the available evidence to the public, which is exemplified in Figure 2, or simply selecting specific parts of the evidence without looking at the big picture (Figure 3). To combat misinformation, it is necessary that experts translate reliable scientific data as clearly as possible to the general public, considering its diverse levels of instruction, with accessible and didactic language in order to combat deleterious beliefs that may negatively impact health if they remain undealt with (7,8,52). Even then, this might unfortunately not be effective enough, since individuals tend to only interpret in a more favorable light information that confirms their beliefs; this universal behavior is based on what is called “confirmation bias” (53).

Moreover, many people find it difficult to judge whether health information comes from a reliable source, or whether it is based on solid scientific evidence or not. This ineptitude exposes people to believe in faulty information, which can lead to detrimental consequences, such as reducing people's engagement in disease screening programs or even lowering adherence to proposed medical treatments (54). Indeed, it is worth stressing that people with less education or less health literacy are the most at risk in this context (55). For instance, one study showed that about 9 out of 10 American adults lack the necessary skills for disease management and prevention (56).

Health literacy is originally defined as the cognitive and social skills that determine an individual's motivation and ability to understand and use information in ways that promote and maintain good health (57). Studies show that people with low health literacy research less about health, choose different sources of information, and have a lower degree of understanding of drug labels and their past health information (57-59). Furthermore, it has also been shown that people with poor health literacy do not trust health information released by government sources and typically determine whether or not health information is reliable based on the position the website is in on search engines or by the quality of its images (60). In contrast, there is evidence indicating that information disclosed by governments and institutions is generally reliable (61,62). A systematic review found that individuals with lower educational levels are generally worse (real and self-rated) at assessing the quality of online health information while also believing information disclosed online more frequently than their more instructed peers (63).

The development of strategies to enhance the dissemination of adequate health orientations in order to protect the public against misinformation is a new area of research. Some interventions against the spread of health-related fake news have already been tested. For instance, it is now possible to use algorithmic and social corrections (i.e., news featuring correct information posted by social network users or presented by an algorithm) in order to protect society against fake news (64). It is also possible to classify the credibility of information sources on social networks (65). Notwithstanding, a truly effective solution is sure to emerge through a combination of diverse points of view made by health, social, and computer science experts working together on interdisciplinary research to find ways to address health misinformation on social media (65-67). It is also necessary to regulate health professionals’ commitment to only transmitting to society information based on the best scientific evidence available while also critically analyzing study results (8).

Considering that coconut oil has already been extensively broadcasted as healthy through several different media and incorporated into people's dietary beliefs as a beneficial fat, it will be a major public health challenge to deconstruct this misconception, in spite of all the available tools to fight misinformation.

In conclusion, the use of coconut oil as a “healthy” component of the western diet is based on the major spread of misconceptions regarding it. The combination of the established knowledge on the negative effects of saturated fats on cardiovascular health and the lack of evidence from clinical trials showing a benefit from coconut oil intake in cardiovascular and metabolic disease advise against the consumption of this oil as a preferential source of dietary fat. Hence, nutritional fat recommendations should be grounded on evidence from robust cardiovascular outcome studies, which is not the case for coconut oil.
